# Standardized patients’ experience of participating in medical students’ education: a qualitative content analysis

**DOI:** 10.1186/s12909-024-05531-x

**Published:** 2024-05-28

**Authors:** Banafsheh Ghorbani, Alun C. Jackson, Nahid Dehghan-Nayeri, Fatemeh Bahramnezhad

**Affiliations:** 1 Nursing department, Student Research Committee, University of Social Welfare and Rehabilitation Sciences, Tehran, Iran; 2 Australian Centre for Heart Health, Melbourne, Australia; 3 Faculty of Health, Deakin University, Geelong, Australia; 4 Centre on Behavioural Health, Hong Kong University, Sandy Bay, Hong Kong, China; 5 Department of Nursing Management, Nursing and Midwifery Care Research Center,School of Nursing and Midwifery, Tehran University of Medical Sciences, Tehran, Iran; 6 Department of Critical Care Nursing, Nursing and Midwifery Care Research Center, School of Nursing & Midwifery, Tehran University of Medical Sciences, Tehran, Iran; 7 School of Nursing and Midwifery, Tehran University of Medical Sciences, Nosrat st, Tohid sq, Tehran, I.IRAN141973317 Iran

**Keywords:** Quality improvement, Simulation, Medical education, Standardized patients, Patient actor, Qualitative research, Content analysis, Iran

## Abstract

**Background:**

Standardized patients are considered a significant educational method in medical sciences and have been successfully employed for many years. This study was conducted with the aim of explaining the experience of standardized patients participating in the education of medical student.

**Method:**

A qualitative content analysis approach was used. This study was conducted at Standardized Patient Center, Tehran University of Medical Sciences, Tehran, Iran, May to February 2022. Fifteen standard patients were selected through purposive sampling with maximum variation. Semi-structured, in-depth, face-to-face interviews were conducted with standard patients. The average duration of the interviews was 60 to 90 min. Data were transcribed and analyzed using the Graneheim and Lundman approach.

**Results:**

A passport for the future and duality of feelings were the two main themes identified in this study with six subthemes. One of the main themes was passport for the future with subthemes creative, participation in educating future generation, reflection and another theme was duality of feeling with subthemes feeling of value, guilty conscience, and fear of judgment by others.

**Conclusion:**

The participants expressed having mixed feelings about their role as standard patients. They felt conflicted because they were compensated for their participation, which made them worry about being judged by others and feel guilty about taking the fee. Therefore, it is recommended to conduct further studies in this area.

## Background

Medical education has evolved over many years, shifting its focus towards simulation-based training. The introduction of simulation into the realm of medical education has transformed the paradigm from lecture-based teaching and evaluation to practice-oriented learning [1]. Today, the ability to communicate effectively with patients and other healthcare professionals has gained significant importance [2]. SPs are people who have received special training to accurately convey the story of a patient in such a way that even experienced healthcare professionals would not be able to distinguish them from real patients [3]. Essentially, they embody all the physical and psychological characteristics of actual patients, providing learners with a holistic understanding of patient care [4]. These individuals are frequently employed in health sciences education to promote the development of communication skills and other essential competencies. They are capable of consistently reflecting a wide range of scenarios [5, 6].

In addition, frequent engagements with SPs accompanied by constructive feedback and self-reflection contribute the continued improvement of students’ performance [7–9]. The use of SPs allows learners to acquire various skills, including motivational interviewing, interpersonal communication, clinical note-taking, collaboration among healthcare professionals, and exposure to uncommon patient scenarios, thus enabling students to interact with patients in a safer environment while broadening their skillset [5, 10].

Apart from the advantages mentioned above, research has shown that utilizing SPs in healthcare education offers numerous benefits. Students exhibit consistently favorable attitudes towards SPs, and their use results in increased learning, improved communication skills, heightened confidence, and better psychomotor performance [5, 11, 12]. Additionally, SPs can serve as an effective tool in augmenting students’ cultural competency training [13]. SPs present a fair and equitable means of evaluating students’ clinical performance since each case repetition is presented to learners in a standardized format [14, 15]. To ensure the optimal utilization of SPs and maximum benefit for students, it is essential for educators to provide adequate support for SPs. This may involve preparing them in areas such as role-play, assessment, and feedback, with faculty supervision playing a significant role. Ultimately, successful implementation of the SPs approach depends on providing appropriate resources and guidance to those involved in the process [16]. Although SPs play a pivotal role in the during the many years that the student is learning, ignoring their requests can lead to complications in their collaboration with students and educators. SPs have various demands that must be addressed to ensure their active participation, such as being heard and understood without judgment by students, teachers, and other team members, as well as receiving empathy and support. It is critical to acknowledge and accommodate the requests of SPs to facilitate effective collaboration and maximize the benefits of their involvement in healthcare education [17, 18]. If the interaction between a student and a service provider fails to consider important factors such as gender ethnicity, and religion, difficulties in establishing a positive and productive relationship may be encountered [19, 20].

The profession of being a standardized patient is still emerging, and gaining a deeper understanding of the people who work in this complex and continually evolving role can help students develop a better understanding of patients and learn important processes in a safe environment. As a result, this contributes to the growth and advancement of SPs as a professional group [1, 21, 22]. The researcher found out about SPs’ concerns and based on that, he decided to find out about their experience of being SPs. In the search, very few studies were found about the experience of these people. The purpose of using SPs is to enhance students’ practical skills and prepare them for real-life clinical situations by providing opportunities for interaction. This study seeks to gain insight into the experiences and perspectives of SPs, with the aim of improving the learning process and its outcomes.

It is important to acknowledge and understand both the visible and hidden aspects of the experiences of individuals involved in the education of medical science students, especially SPs. Qualitative research is an effective method for identifying and exploring these issues. The purpose of this study was to explore the experiences of SPs who participate in the education of medical science students.

## Method

### Study design

In this qualitative study, Graneheim and Lundman’s (2004) [23] conventional content analysis approach was used to explain the experiences of SPs. This is further explained in the following section on data analysis.

### Participants

A total of fifteen SPs from the Standardized Patient Center, Tehran University of Medical Sciences, Tehran, Iran consented to take part in the study who were eligible to participate. Participants were purposeful selected to take part in the study. The first of participants was recommended to the researcher by the head of the SP center. Inclusion criteria consisted of willingness to participate, ability to communicate experiences in Farsi, and a minimum of 6 months’ experience as a SP.

### Data collection

Key informants were purposefully sampled from among the SPs collaborating with the Tehran University of Medical Sciences Research and Development Center, Tehran, Iran, May to February 2022. The objectives and methods of the study were explained to the participants, including ensuring them of the confidentiality of their information. Written and verbal informed consent was obtained from each participant before their participation in the study. In-depth, semi-structured interviews were conducted at a time and place arranged according to the participant’s request. Most interviews took place in the corresponding author’s office, while two interviews were conducted at the Clinical Skills Center as per the participant’s preference. All the researchers involved in this study conducted the review and analysis of the interviews. The interviews continued until data saturation was achieved, meaning that no new information or codes emerged from the last two interviews. During the analysis of the final interviews, it was found that no additional data was included in the study that would lead to the development, modification, enlargement, or addition to the existing theory. As a result, no new category appeared and the categories remained unchanged. The primary focus of the interviews revolved around the individuals’ experiences as SPs in the student education process, so the interviews commenced with the question: “What is your experience as an SP in the student education process?” What does he think when he uses you as an SP? What are your challenges? Probing questions were used to clarify concepts based on the information provided by the participants. Each interview lasted approximately 60 to 90 min. The interviews were conducted in Persian. They began with warm-up questions, followed by the main questions and probing inquiries. At the end of each interview, participants were asked if there were any remaining topics they wished to discuss, and were thanked for their participation.

### Data analysis

The qualitative content analysis approach developed by Graneheim and Lundman (2004) [23] was employed to analyze the data. The process involved several steps, including identifying meaning units, condensing them, assigning codes, establishing subthemes and themes, and identifying overarching themes. The interviews were transcribed with each transcription being thoroughly reviewed multiple times to gain a comprehensive understanding of the content. Subsequently, a line-by-line analysis was conducted to identify the meaning units, which were then condensed and labeled with appropriate codes. These codes were further grouped into subthemes based on their similarities and differences. By bringing similar subthemes together, broader themes were formed, ultimately leading to the emergence of thematic patterns that conveyed the underlying meaning within the text. Throughout this process, all the researchers conducting the fieldwork engaged in discussions and evaluations of the themes and subthemes.

It is important to note that both explicit content analysis and latent content analysis were conducted in this process to extract the final themes. Additionally, constant comparison analysis, a key approach in qualitative text analysis, was utilized. The extracted themes and sub-themes were consistently compared and reviewed with each other and with the written narratives.

The first author conducted and transcribed the interviews. She then conducted the coding, which was later reviewed by the first author. In cases of conflicting opinions, the second and third authors would share their perspectives and collectively decide on the codes.

### Rigor

The study’s rigor was evaluated using Lincoln and Guba’s criteria [24], which consists of credibility, dependability, confirmability, transferability, and authenticity.

Credibility : the interview text and codes were shared with participants for feedback on accuracy and validity. Any discrepancies were addressed and investigated. Additionally, the researcher clarified unclear cases or misunderstood participant meanings through phone calls and emails.

Dependability: all interviews were recorded and transcribed, and the coding and data analysis were reviewed by the entire research team. Additionally, an external professor evaluated a subset of interviews to ensure the quality of coding.

Confirmability: it was ensured by providing a comprehensive description of the data, allowing external observers to evaluate and understand the research process. Fourthly, transferability was enhanced by employing maximum variation sampling, accurately describing the participants, sampling methods, and the time and location of data collection. This increased the potential applicability of the findings to other contexts.

Transferability: it was addressed by obtaining informed consent from all participants, fostering trust, clarifying the research method to participants and the audience, and making the research report available to both respondents and the wider audience.

### Ethical considerations

The current study was approved with ethics code (IR.TUMS.MEDICINE.REC.1400.1346) by the Ethics Committee at Tehran University of Medical Sciences. The objectives and methods used in the study were fully explained to the participants, assuring them about the confidentiality of the shared information and maintaining their anonymity. Additionally, the aim of using audio recording was explained to the participants, and they were reassured that participation in the study is optional, with the freedom to withdraw at any time.” Also, written and verbal informed consent was obtained from each participant before their participation in the study.

## Results

Based on the demographic characteristics, the mean age ± sd of the participants in this study was 41.23 ± 16.8. The age range of the participants in the study was 16–66 years. Other demographic characteristics are listed in Table 1.

**Table 1:** Demographic characteristics of study participants

Demographic Characteristics		Frequency	Percentage (%)
**Marital Status**	Single	3	20
	Married	10	66.67
	Widow or divorced	2	13.33
**Employment Status**	Student	1	6.6
	Not employed outside the home	7	46.66
	Employed	5	33.33
	Retired	2	13.33
**Sex**	Female	10	66.67
	Male	5	33.33
**Educational Level**	High School	11	73.33
	University graduate	2	13.33
	no formal education	2	13.33
**Duration of Being SPs/year**	1≥	1	6.6
	1–5	13	86.66
	5≤	1	6.6

Based on the analysis of the data two themes were emerged, “Passport for the Future” and “Duality of Feeling” with six subthemes (Table 2).

**Table 2:** Themes and sub-themes that appeared in the study

Themes	Subthemes
Passport for the future	Creative Participation in educating future generation Reflection
Duality of feeling	Feeling of value Guilty conscience Fear of judgment by others

### Passport for the future

Passport for the Future, consisted of three subthemes: “Creativity,” “Participation in Educating Future Generations,” and “Reflection.” The participants emphasized their responsibility in creating an environment that enables medical science students to develop confidence in their skills before they enter the clinical setting. They believed that their activities were essential in ensuring the future success of these students as healthcare professionals. By using patient simulators, students could reflect on their actions, make decisions, and adjust their approaches without the pressure or fear of causing harm to real patients. This enabled students to take breaks from practical work, review their activities, engage in discussions with teachers and peers, and then continue their tasks. The participants considered their role in educating students as crucial and foundational, providing a stepping stone for their progression in clinical education. Collaborating with these participants allowed students to effectively handle various scenarios, utilizing the ample time available for thinking and even exploring innovative strategies in managing the clinical environment.

#### Creativity

The participants in the study indicated that interacting with a real person who can communicate effectively motivates individuals to go above and beyond to help solve problems. This interaction also provides students with the opportunity to exercise critical thinking skills, fostering creativity and enabling them to find logical and accurate solutions to their issues.

Participant number 3, who had been acting as a patient simulator for two years and three months, stated:

“*I’ve seen many times that the student has enough time to learn how to manage the environment with their own approach. For example, they learn how to speak with a patient who has a taboo disease. I remember a student came to talk to me when I was playing the role of a patient with AIDS, to talk and take a history. I remember I was supposed to try to hide my disease. The student was persistent at first, using everything they knew to get me to talk, but was not successful. In the end, they figured out what to say to me to make me open up.”*

#### Participation in educating future

The participants emphasized that they play a crucial role in evaluating students, assisting clinical professors in determining whether a student should progress to a higher level based on their performance in interactions with SP. They viewed their own performance as integral to the student’s advancement and competence assessment, believing that without their input, professors may struggle to make accurate judgments about a student’s abilities. They asserted that they are among the key decision-makers involved in determining a student’s promotion and qualification.

“*I’m happy because if a student passes me, they can get a passing grade*,“(Participant 8).

#### Reflection

In fact, the participants believed that when a student interacts with them in the process of education and evaluation, their stress levels is high. This is because it is not like the clinical environment where the person knows they are interacting with a patient. On the other hand, the patient is seen as a human being and not just a mannequin that does not interact back. Therefore, the student is placed on a path where they have the opportunity to rethink their performance, calmly assess it, and review it. When the student interacts with the patient and observes their reaction, they can reflect on their own way of dealing with a human being, which aids in their learning.

In this regard, one of the participants shared the following experience:

“*One day, a student entered the room and did not greet me. He immediately started reading from a script and bombarding me with questions. I simply looked at him for a few moments and then responded to his questions with complete indifference. I didn’t even make eye contact with him. It’s worth mentioning that my behavior was intentional. I wanted to teach the student that their patient is a human being and they should respect this relationship. By establishing a good rapport, you can gather valuable information from the patient.” The student looked at me and said, “I’m sorry, I didn’t greet you. I now realize my behavior was inappropriate*.“(Participants 11).

### Duality of feeling

“Duality of Feeling,” encompassed three subthemes: “Feeling of Value,” “Guilty Conscience,” and “Fear of Judgement by Others.” Participants expressed conflicting emotions within this theme. On one hand, they felt a sense of importance and value when considering their role in facilitating students’ learning and supporting their educational journey. However, there was also a prevailing feeling of guilt because participants received payment for their involvement in the educational process. They believed that as educators, they should contribute without monetary compensation. Nonetheless, financial needs compelled them to accept payment for their services. Furthermore, participants shared concerns about the perception others might have if they found out about receiving payment. They feared being seen as financially needy or from a lower social class, leading to a diminished sense of worth. Consequently, they preferred to keep their involvement and compensation a secret. The worry about how students perceived them consistently occupied their thoughts.

#### Feeling of value

The feeling of being a valuable member of the teaching process is important to them.They believe that their involvement in education medical, nursing, and midwifery students is significant. Knowing that they are contributing to the development of future healthcare professionals and ultimately helping patients makes them feel valued and appreciated.

On the other hand, participant number 10, a 21-year-old woman who had been working as a standard patient for three years, remarked:

“*It’s a great feeling to know that you’re contributing to something. I always think that I have as much right to the students as the teacher does. When I play the role of a patient, it helps the information stick better in their minds, and this gives me confidence.”*

#### Fear of judgment by others

One of the concerns expressed by participants in this study was the fear of being judged by others. They mentioned that receiving money for this work might lead others to believe that they are financially needy individuals who are allowing themselves to be examined or play certain roles. This perception made them feel conflicted, as they did not want anyone to know about their participation in this activity. In fact, they preferred to keep this aspect of their lives hidden. At times, they even feared that their peers would view them differently, leading to negative emotions.

In this regard, participant number 11, who was 66 years old and entered this process after retirement, said:

“When I retired, due to the respiratory problem I had and the fact that I also have rheumatism, I couldn’t do heavy work. On the other hand, my retirement pension was not enough. My niece, who was in this line of work, told me about it but asked me not to tell anyone, as they would think I entered this line of work out of misery.”

#### Guilty conscience

They expressed a desire to be involved in the education process, but due to their circumstances, they receive payment for their participation. They believe that education is a sacred act and should not involve financial compensation. They would prefer to receive a salary, but their economic situation forces them to accept payment, which makes them feel guilty.

Participant number 2 shared their thoughts on the matter, stating:

“*I always feel uneasy about being paid to teach students. But I have no choice due to my financial needs. If I were in a better financial position, I would never accept payment because education is a shared responsibility and should not be monetized. The satisfaction of educating others is rewarding enough for me. I don’t want money to be a factor in how I live my life, and it troubles me*.”

## Discussion

In interpreting the experience of SPs participating in the education of medical science students, two main themes with six subthemes were revealed. “A Passport to the Future,” with subthemes “Creativity,” “Participation in Nurturing the Next Generation,” and “Reflection” was emerged in this study, Participants stated that the work they do serves as a bridge for students to reach a brighter future, and they see themselves as playing a crucial role in this educational approach.

According to a study conducted by Peisachovich et al. in 2016, the utilization of SPs has been found to enhance various skills such as communication, idea exchange, clinical expertise, and management abilities. This study also highlighted the positive impact of SPs on reflection and competence development [25]. Similarly, Lashley et al. in 2009 demonstrated that the use of SPs contributes to improved problem-solving, decision-making, and scene management skills [26]. Another study by Kameg et al. in 2010 confirmed the effectiveness of SPs in enhancing communication skills [27]. Doolen et al.‘s research in 2014 revealed that student skills and learning related to real patient interactions were significantly enhanced through the use of SPs [28]. Okinyi et al. in 2022 observed that targeted communication with patients, proper interviewing techniques for HIV patients, and effective management and evaluation of the environment were achieved through the use of SPs [29]. Blumling et al.‘s study in 2018 focused on the initial assessment of partner satisfaction. It concluded that SPs increased students’ proficiency in communication, history taking, and knowledge needed for real-world healthcare scenarios [30]. Slater et al. in 2016 found that the utilization of SPs not only boosted students’ self-confidence but also had a significant impact on cognitive, emotional, and psychomotor learning. Students expressed that practicing with SPs better prepared them for real-world patient encounters and enhanced their performance [31]. Burrell et al.‘s study in 2021 involving nursing students reported high levels of satisfaction, self-confidence, and preparedness for clinical practice when using SPs [32]. An earlier study found that the use of SPs significantly reduced students’ anxiety and improved their scene management skills, ultimately enhancing their ability to provide care [33].

As with the present study, that study identified a duality of feelings among SPs, including a sense of value, guilt, and fear of judgment from others. SPs create a safe and supportive environment where students can apply theoretical and clinical skills. Jarosinski et al.‘s study in 2016 revealed that fulfilling the role of SPs instilled a sense of value, joy in goal-oriented actions, and a feeling of being part of the educational team. SPs believed they could provide valuable training to students as mentors [34]. However, Fluet et al.‘s study in 2022 highlighted that SPs became aware of students’ judgments and prejudices when playing certain roles related to sexual orientation or drug addiction. Factors such as gender and ethnicity also influenced their experiences as SPs, and organizational biases contributed to a fear of judgment [35]. The use of SPs in curricula has been widely recognized for creating a dynamic learning environment. It allows instructors to introduce realistic scenarios aligned with learning objectives, control the learning environment, provide feedback, and facilitate the integration of theory and practice through guided explanation and reflection processes [25]. The use of SPs effectively simulates various clinical problems that students may encounter, providing a safe space for learning.

### Implications for practice

A crucial implication of these findings for clinical practice is the necessity of providing students with adequate education to ensure safe patient care. By providing support to SPs and empathically addressing their needs and emotions, healthcare professionals can deliver improved services. Moreover, SPs play a pivotal role in offering educational services to students, aiding in their optimal learning and development.

### Recommendations

It is crucial to emphasize the importance and preserve the human dignity of individuals who participate as SP in educational settings. This approach benefits both the SPs and the students involved in the learning process. To begin with, educating SPs about their significant role helps them understand that they are valued contributors within the education system. By emphasizing their importance, they can develop a sense of fulfillment and purpose in helping train future healthcare professionals. Recognizing their impact can also boost their confidence and motivate them to actively engage in the educational process. It is also equally vital to educate students about the value and significance of the SPs ‘s role. Students should be taught to appreciate and respect the SPs as an essential part of their learning experience. This includes understanding that the SPs ‘s participation and reflection contribute to their growth as healthcare practitioners. Teaching students to respect the SPs involves fostering empathy, communication skills, and professionalism. They should learn how to interact with the SPs in a compassionate and respectful manner. This includes active listening, showing empathy, seeking consent, maintaining privacy and confidentiality, and treating the SPs with dignity throughout their interactions. Furthermore, incorporating reflective exercises and discussions into the curriculum can help students recognize the perspectives and experiences of SPs. This cultivates a deeper understanding and appreciation of the challenges they may face while participating in medical education. By emphasizing the importance of the SPs’s role and teaching students to respect and value their contributions, we establish a positive and inclusive learning environment. This approach promotes empathy, professionalism, and ethical behavior among students, preparing them to provide high-quality care while upholding the dignity and well-being of all patients they encounter in their future career.

### Limitation

One of the most significant limitations of this study was the unwillingness of the SPs to be interviewed. They believed that sharing their experiences could increase negative perceptions towards them. In this context, they were reassured that their information would remain entirely confidential and they agreed to be interviewed. Also, despite the researcher’s efforts, three individuals who played the roles of people with sexual problems were not willing to be interviewed under any circumstances. On this basis, it is recommended that researchers design studies on the experiences of SPs who play roles with sexual disorders. Also, studies should be designed to enhance their role and provide support for them.

## Conclusion

The studies highlight the important role of incorporating SPs in healthcare education due to their numerous advantages in terms of skill development, confidence building, anxiety reduction, scene management improvement, and the creation of a realistic learning environment. Over the past few decades, the use of SPs in the healthcare industry has greatly expanded for skill development and assessment purposes. These simulated patients now play diverse roles, representing various characteristics found in the patient population such as different ages, genders, socio-economic backgrounds, physical and mental illnesses, and distinct personality types. It is crucial to have awareness on how to effectively utilize this method and collaborate with SPs. This involves providing systematic and well-planned education, ensuring successful outcomes for teachers, coaches, and SPs actors. Furthermore, SPs also serve as expert educators, emphasizing the need to consider their work environment and satisfaction with participation in education programs. Developing accurate clinical scenarios based on theory, implementing systematic education programs focused on the role and function of SPs, creates favorable conditions for employing this approach. Ultimately, this contributes to enhancing professional competence and improving the quality of clinical evaluation within the medical and health sciences field.

During the interviews, no findings were found that cultural or gender differences (considering the context of Iran being Islamic) cause a different experience in standardized patients (SPs). Regarding the use of SPs at Tehran University of Medical Sciences, the process is as follows: if a professor makes a written request, the desired SP will be introduced to the standardized patient center of the university, and the scenarios will be explained to the SP by the professor. For each scenario, the SP is charged a fee by the standardized patient center. SPs in Iran do not have a fixed salary, so it is suggested that these individuals should be considered for a job with a fixed salary.

## Data Availability

This study are not publicly available due to their qualitative nature but are available from the corresponding author on reasonable request.
